# Differentiation of Bos grunniens and Bos taurus based on STR locus polymorphism

**DOI:** 10.18699/VJGB-23-59

**Published:** 2023-09

**Authors:** K.B. Chekirov, Zh.T. Isakova, V.N. Kipen, M.I. Irsaliev, S.B. Mukeeva, K.A. Aitbaev, G.A. Sharshenalieva, S.B. Beyshenalieva, B.U. Kydyralieva

**Affiliations:** Kyrgyz-Turkish Manas University, Bishkek, Kyrgyz Republic; Research Institute of Molecular Biology and Medicine, Bishkek, Kyrgyz Republic; Institute of Genetics and Cytology of the National Academy of Sciences of Belarus, Minsk, Republic of Belarus; Research Institute of Molecular Biology and Medicine, Bishkek, Kyrgyz Republic; Research Institute of Molecular Biology and Medicine, Bishkek, Kyrgyz Republic; Research Institute of Molecular Biology and Medicine, Bishkek, Kyrgyz Republic; Kyrgyz State University named after I. Arabaev, Bishkek, Kyrgyz Republic; Kyrgyz State University named after I. Arabaev, Bishkek, Kyrgyz Republic; Kyrgyz-Turkish Manas University, Bishkek, Kyrgyz Republic

**Keywords:** domestic yak, Bos grunniens, cattle, Bos taurus, DNA, microsatellite markers, STR, genotyping, differentiation, домашний як, Bos grunniens, крупный рогатый скот, Bos taurus, ДНК, микросателлитные маркеры, STR, генотипирование, дифференциация

## Abstract

Differentiation of closely related biological species using molecular genetic analysis is important for breeding farm animals, creating hybrid lines, maintaining the genetic purity of breeds, lines and layering. Bos grunniens and Bos taurus differentiation based on STR locus polymorphism will help maintain the genetic isolation of these species and identify hybrid individuals. The aim of this study is to assess the differentiating potential of 15 microsatellite loci to distinguish between domestic yak (B. grunniens) bred in the Kalmak-Ashuu highland region (Kochkor district, Naryn region, Kyrgyz Republic) and cattle (B. taurus) of three breeds (Aberdeen-Angus, Holstein and Alatau) using molecular genetic analysis. The samples were genotyped at 15 microsatellite loci (ETH3, INRA023, TGLA227, TGLA126, TGLA122, SPS115, ETH225, TGLA53, BM2113, BM1824, ETH10, BM1818, CSSM66, ILSTS006 and CSRM60). Twelve of the loci were from the standard markers panel recommended by ISAG. Statistical analysis was performed using GenAlEx v.6.503, Structure v.2.3.4, PAST v.4.03, and POPHELPER v1.0.10. The analysis of the samples’ subpopulation structure using the Structure v.2.3.4 and 15 STR locus genotyping showed that the accuracy of assigning a sample to B. taurus was 99.6 ± 0.4 %, whereas the accuracy of assigning a sample to B. grunniens was 99.2 ± 2.6 %. Of the 15 STRs, the greatest potential to differentiate B. grunniens and B. taurus was found in those with the maximal calculated FST values, including BM1818 (0.056), BM1824 (0.041), BM2113 (0.030), CSSM66 (0.034) and ILSTS006 (0.063). The classification accuracy of B. grunniens using only these five microsatellite loci was 98.8 ± 3.4 %, similar for B. taurus, 99.1 ± 1.2 %. The proposed approach, based on the molecular genetic analysis of 5 STR loci, can be used as an express test in Kyrgyzstan breeding and reproduction programs for B. grunniens

## Abstract (ru)

Дифференциация близкородственных биологических видов с использованием методов молекулярно-генетического анализа имеет важное значение для селекционных процессов при разведении сельскохозяйственных животных, создании гибридных линий, поддержании генетической чистоты пород, линий, отводок. Подход к дифференциации Bos grunniens и Bos taurus на основании полиморфизма STR-локусов будет способствовать поддержанию генетической обособленности данных видов и, как следствие, выявлению гибридных особей. Целью исследования была оценка дифференцирующего потенциала 15 микросателлитных локусов для различения особей домашнего яка (B. grunniens), разводимых в высокогорном регионе Калмак-Ашуу (Кочкорский район, Нарынская область, Кыргызская Республика), и крупного рогатого скота (B. taurus) трех пород (абердин-ангусская, голштинская и алатауская) с использованием молекулярно-генетического анализа. Образцы были генотипированы по 15 микросателлитным локусам (ETH3, INRA023, TGLA227, TGLA126, TGLA122, SPS115, ETH225, TGLA53, BM2113, BM1824, ETH10, BM1818, CSSM66, ILSTS006 и CSRM60). Двенадцать из рассматриваемых STR-локусов составляли стандартную панель маркеров, рекомендованную ISAG. Статистический анализ данных проводили с использованием программ GenAlEx v.6.503, Structure v.2.3.4, PAST v.4.03 и POPHELPER v1.0.10. Анализ субпопуляционной структуры исследуемых выборок в программе Structure v.2.3.4 по данным генотипирования 15 STR-локусов показал, что точность отнесения образца к B. taurus составила 99.6 ± 0.4 %, к B. grunniens – 99.2 ± 2.6 %. Наибольшим потенциалом для дифференциации B. grunniens и B. taurus обладали те локусы, для которых рассчитанные значения показателя FST оказались максимальными – BM1818 (0.056), BM1824 (0.041), BM2113 (0.030), CSSM66 (0.034) и ILSTS006 (0.063). Точность классификации B. grunniens с использованием только этих пяти микросателлитных локусов составила 98.8 ± 3.4 %, B. taurus – 99.1 ± 1.2 %. Предложенный нами подход, основанный на молекулярно-генетическом анализе пяти STR-локусов, может быть использован в качестве экспресс-теста в селекционных и воспроизводительных программах Кыргызстана для B. grunniens

## Introduction

Kyrgyzstan is characterized by a variety of natural and climatic conditions, therefore, animal husbandry may vary a lot between locations. Thus, breeding yaks at high altitude makes much sense given that the natural conditions are favorable for the species. In contrast, breeding cattle develops more at low and middle altitudes. Compared to cattle, yaks use lowgrowth pasture feed better, and in winter they extract it from under a snow cover 10–15 cm thick. Yak meat is not inferior to beef and is rich in proteins, as well as trace elements vital for humans. Although yak milk output is low, their milk is known for the high content of fat (5.5–8.6 %), phosphorus (0.28 %) and calcium (0.30 %) (Abdykerimov, 2001). The yak not only produces milk, meat, skin and wool, but is also used for transport by people in the highlands of Asia (Chertkiev, Chortonbaev, 2007). Yak is a very strong alternative for domestic cattle, easy to breed at high altitude with a very harsh and cold climate. Yaks have thick subcutaneous fat, covered with thick long hair, as well as sharp “steel” hooves that allow them to move along very steep, rocky trails, unattainable for any other livestock.

Unlike the common cattle, which is currently bred on all continents, domestic yak has a very small geographical distribution area, which is limited to the mountainous regions of Central Asia (Jacques et al., 2021). The reason for this, according to (Luz, 1936), is the animal itself. Domestic yak, as well as its wild relative, the Tibetan yak, is perfectly adapted to the conditions of high altitude and mountain plateaus (Lyz, 1936). They both live in the harsh climate of the highlands, where the annual temperature is close to zero for more than eight months a year, and the minimal temperature can drop to –50 °C. In such harsh conditions, yaks live all year round in the open air on pasture

One of the ways to further intensify yak breeding as an independent branch of animal husbandry is to improve breeding technology, yak breeding and productive qualities, expand knowledge on their biology, as well as increase meat productivity. The study of yak genetic characteristics allowing them to live in the harsh climate of the highlands is of great practical interest

Currently, the most convenient genetic markers describing genetic structure of different animal species, including yaks and cattle, are polymorphic microsatellite DNA loci (STR, short tandem repeat), which have a codominant nature of inheritance and serve as an indispensable tool to study genetic differences not only between animals, but also populations of the same breeds, as well as between breeds.

The aim of this study is to evaluate the differentiating potential of 15 STR loci (ETH3, INRA023, TGLA227, TGLA126, TGLA122, SPS115, ETH225, TGLA53, BM2113, BM1824, ETH10, BM1818, CSSM66, ILSTS006 and CSRM60) to distinguish individuals of Bos grunniens and Bos taurus.

## Materials and methods

The biological material for molecular genetic research was blood samples taken from adult livestock, including 55 domestic yaks (B. grunniens) bred in the Kalmak-Ashuu highland region (Kochkor district, Naryn region, Kyrgyz Republic), which comprised a sample called YAK, as well as blood DNA samples taken from an adult herd of 145 cows (B. taurus) of three breeds, including Aberdeen-Angus (n = 45, sample ABR), Holstein (n = 50, sample HOL) and Alatau (n = 50, sample ALA). All applicable international, national and/or institutional principles for the care and use of animals have been observed.

DNA isolation was carried out by phenol-chloroform extraction (Sambrook, Russell, 2001). The samples were genotyped by 15 microsatellite loci. Of the analyzed STR loci, 12 were the standard markers panel recommended by the International Society of Animal Genetics (ISAG), including ETH3, INRA023, TGLA227, TGLA126, TGLA122, SPS115, ETH225, TGLA53, BM2113, BM1824, ETH10 and BM1818. Microsatellite loci CSSM66, ILSTS006 and CSRM60 were analyzed additionally. Oligonucleotides sequence is shown in Table 1.

**Table 1.**
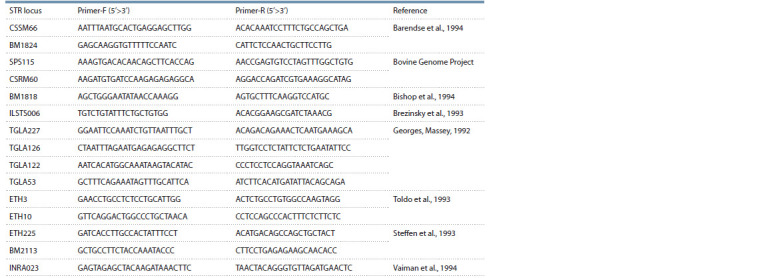
Oligonucleotides sequence for 15 STR loci

PCR was analyzed using capillary electrophoresis via an automatic genetic analyzer with a laser-induced fluorescence detection Applied Biosystems 3500 (ThermoFisher, USA). Samples validated using the COrDIS Cattle kit (LLC “GORDIZ”, Russian Federation) were used as a reference for allelic calculation

Statistical analysis was carried out using GenAlEx v.6.503 (Peakall, Smouse, 2012), Structure v.2.3.4 (Pritchard et al., 2000), PAST v.4.03 (Hammer et al., 2001) and POPHELPER v1.0.10 (Francis, 2016). GenAlEx v.6.503 was used to estimate genetic distances using the AMOVA (analysis of molecular variation) method; Structure v.2.3.4 was used to calculate the Q criterion, which characterizes the attribution of each individual to the corresponding cluster (subgroup within the group); POPHELPER v1.0.10 web application was utilized for graphical interpretation of results obtained in Structure v.2.3.4, whereas PAST v.4.03 was used to plot the main components based on the calculation of genetic distances using the AMOVA method.

## Results and discussion

**Fig. 1.**
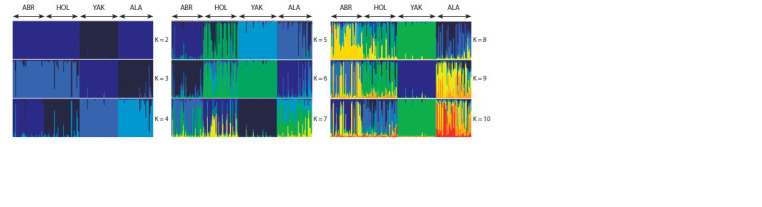
Genetic structure of the studied samples for the most probable cluster count (K) from 2 to 10. ABR – Aberdeen-Angus, HOL – Holstein, YAK – domestic yaks, ALA – Alatau.

The analysis of the subpopulation structure of B. grunniens and B. taurus using the Structure v.2.3.4 program on the genotyping data of 15 STR loci, as well as the graphical representation showing the assignment of individuals to a specific group, produced by the POPHELPER v1.0.10 web application, is shown in Figure 1.

As a result of the simulation (the duration of the burn-in 5000, the number of MCMC (Markov chain Monte Carlo) repetitions after the burn-in 50,000, 10 iterations), we found four distinct clusters (K = 4, ΔK = 83.2). Structure v.2.3.4, according to the method of J.K. Pritchard (Pritchard et al., 2000), allowed to compute the Q criterion, which characterized the assignment of each individual to a group (species) for four samples, including ABR, HOL, ALA and YAK. Q ≥ 75 % in the ABR sample was found in 88.9 % (40/45) individuals, accuracy 94.4 ± 5.7 %; HOL, in 82.0 % (41/50), accuracy 95.8 ± 3.3 %; ALA, in 90.0 % (45/50), accuracy 96.3 ± 4.3 %; and YAK, in 98.2 % (54/55), accuracy 98.3 ± 3.2 %. When combining three B. taurus samples into one (COW) and analyzing only two groups – COW and YAK, Q ≥ 75 % was identified in 100 % (145/145) individuals, accuracy 99.6 ± 0.4 % in the first group and in 98.2 % (54/55), accuracy 99.2 ± 2.6 % in the second group

**Fig. 2.**
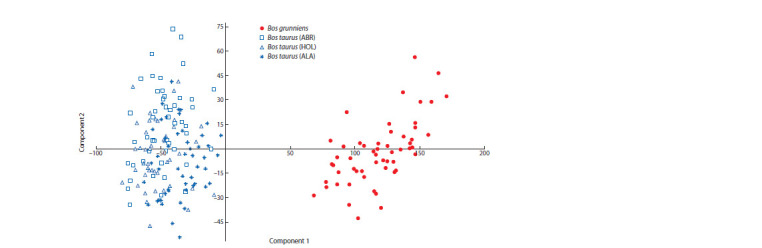
Principal component analysis (all 15 STR loci).

Based on the genetic distances analysis calculated using the AMOVA algorithm, we constructed a graph of principal component analysis (PCA) (Fig. 2). COW and YAK groups on the graph are spaced relative to each other and form two non-overlapping arrays.

Of all 15 STR loci analyzed in the study, including ETH3, INRA023, TGLA227, TGLA126, TGLA122, SPS115, ETH225, TGLA53, BM2113, BM1824, ETH10, BM1818, CSSM66, ILSTS006 and CSRM60, those with the highest calculated FST had the greatest potential to differentiate B. grunniens and B. taurus (Table 2).

**Table 2.**
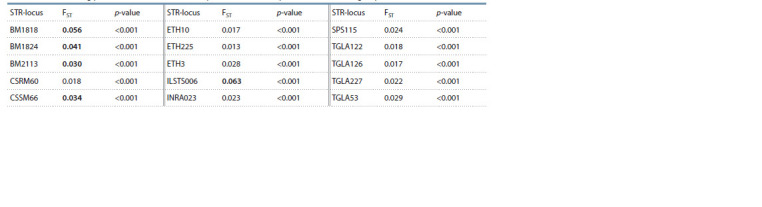
Differentiating potential of 15 STR loci (locus-by-locus AMOVA analysis for COW and YAK groups) Values in bold are greater than 0.03.

A similar approach aimed to develop an algorithm to differentiate evolutionarily close animals using STR loci was described in (Rębała et al., 2016; Nosova et al., 2020).

The highest calculated FST values are shown for BM1818, BM1824, BM2113, CSSM66 and ILSTS006 STR loci. Table 3 summarizes the allelic diversity and allele frequency for the STR loci listed above.

**Table 3.**
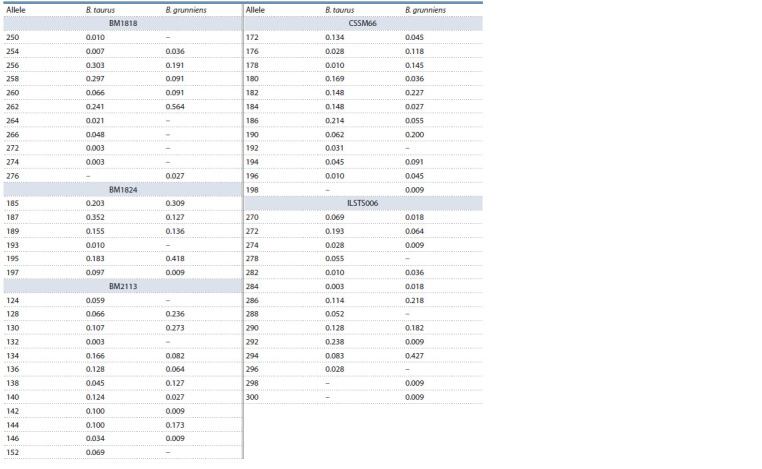
The allele frequency of five STR loci with the highest differentiating potential according to FST values for B. grunniens and B. taurus

As a result, the representation of major alleles was very different between COW and YAK groups. In particular, ‘256’, ‘258’ and ‘262’ (the total frequency of prevalence was 84.1 %) were the major alleles for BM1818 in the COW group, whereas ‘262’ (occurrence 56.4 %) was major for the YAK group. For BM1824, the difference in the frequency of ‘195’ allele in two groups was 24.0 % (COW – 18.3 %, YAK – 41.8 %), and 22.0 % (COW – 35.2 %, YAK – 12.7 %) for ‘187’ allele. The most common alleles for the BM2113 STR locus in the YAK group were ‘128’ (23.6 %) and ‘130’ (27.3 %), while total frequency of these alleles in the COW group was only 17.2 %.

A similar trend was observed for the CSSM66 locus, and there was a significant difference in the frequency of ‘172’, ‘178’, ‘180’, ‘184’ and ‘190’ alleles. ‘294’ (42.7 %) was the most common allele in the ILSTS006 locus for the YAK group whereas ‘286’ (11.4 %), ‘290’ (12.8 %), ‘272’ (19.3 %) and ‘292’ (23.8 %) were the most prevalent in COW. Table 4 summarizes private alleles for these STR loci.

**Table 4.**
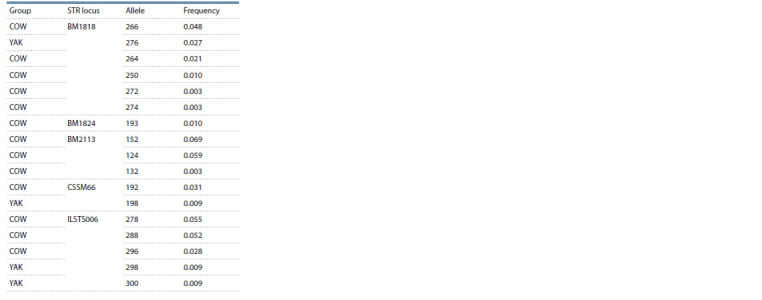
Private allele frequency for COW and YAK

Based on the data obtained, a repeated analysis of the subpopulation structure was completed using Structure v.2.3.4 only for 5 out of 15 STR loci, as a result of genotyping analysis (Table 5). Table 3 presents allelic diversity and allele prevalence for these STR loci.

**Table 5.**
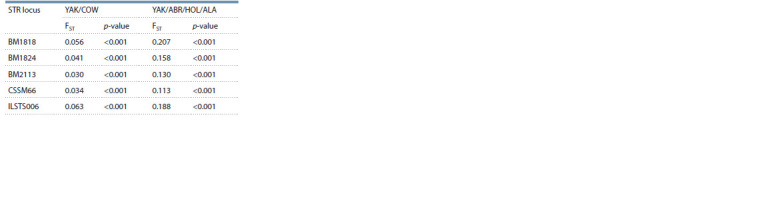
Differentiating potential of five STR loci (locus-by-locus AMOVA analysis)

As a result of the simulation with Structure v.2.3.4 (the duration of the burn-in 5000, the number of MCMC repetitions after the burn-in 50,000, 10 iterations), we found four distinct clusters (K = 4, ΔK = 119.7). Structure v.2.3.4, according to the method of J.K. Pritchard (Pritchard et al., 2000), allowed to compute the Q criterion, which characterized the assignment of each individual to a group (species) for four samples, including ABR, HOL, ALA and YAK. Q ≥ 75 % in the ABR sample was found in 88.9 % (40/45) of the individuals, accuracy 92.6 ± 5.8 %; HOL, in 68.0 % (34/50), accuracy 92.4 ± 6.2 %; ALA, in 82.0 % (41/50), accuracy 93.2 ± 5.6 %; and YAK, in 96.4 % (53/55), accuracy 97.7 ± 3.4 %. To improve the accuracy of individuals differentiation in the Holstein breed, the list of analyzed STR loci should be further expanded, starting with ETH3, TGLA126 and TGLA122.

In total, differentiation accuracy based on BM1818, BM1824, BM2113, CSSM66 and ILSTS006 STR loci in YAK (B. grunniens) was 98.8 ± 3.4 %, and 99.1 ± 1.2 % in COW (B. taurus). Thus, differentiation accuracy was not lost even when 5 STR loci out of 15 were analyzed.

Earlier, Inter Simple Sequence Repeats of yak-cattle hybrids were studied at the Institute of General Genetics RAS, and a species-specific pattern of eight ISSR fragments for yak was found in yak and F1 hybrids populations (Stolpovsky et al., 2014). Also, the allele depository of yaks and their hybrids with B. taurus was assessed earlier using microsatellite analysis, yielding high genetic diversity for F1 hybrids in comparison with the original species (Al-Kaisy, 2011). Our study did not confirm hybrid individuals of B. grunniens and B. taurus.

## Conclusion

This study assessed the differentiating potential of 15 STR loci, including ETH3, INRA023, TGLA227, TGLA126, TGLA122, SPS115, ETH225, TGLA53, BM2113, BM1824, ETH10, BM1818, CSSM66, ILSTS006 and CSRM60 for B. grunniens and B. taurus individuals, as well as attempted to identify hybrids of these species

According to the subpopulation structure analysis, following genotyping of 15 STR loci, the classification accuracy of B. grunniens individuals was 99.1 ± 1.2 %, and 99.6 ± 0.4 % for B. taurus. When the number of STI loci used for decision was limited to five, including BM1818, BM1824, BM2113, CSSM66 and ILSTS006, the differentiating potential of which, according to FST, was the greatest and varied from 0.030 to 0.063, the classification accuracy for B. grunniens was 98.8 ± 3.4 %, and 99.1 ± 1.2 % for B. taurus

Thus, we conclude that the analysis of even a small number of STR loci allows to ascertain differentiation of domestic yak and three breeds of cattle (Aberdeen-Angus, Holstein and Alatau) bred in Kyrgyzstan. At the same time, further research is needed in the longer run to more accurately classify differentiation potential for selected loci.

## Conflict of interest

The authors declare no conflict of interest.
